# Clustering of Health and Oral Health-Compromising Behaviours in Army Personnel in Central Peninsular Malaysia

**DOI:** 10.3390/healthcare11050640

**Published:** 2023-02-21

**Authors:** Ahmad Asyraf Azil, Zamros Yuzadi Mohd Yusof, Jamaludin Marhazlinda

**Affiliations:** 1 Department of Community Oral Health and Clinical Prevention, Faculty of Dentistry, Universiti Malaya, Kuala Lumpur 50603, Malaysia; 2 Dental Services Section, Health Services Division Malaysian Armed Forces, Kuala Lumpur 50634, Malaysia

**Keywords:** clustering behaviour, health-compromising behaviours, oral health-compromising behaviours, adults, military, army

## Abstract

Health- and oral health-compromising behaviours (HOHCBs) impact the health readiness of military personnel, resulting in decreased fitness performance, thus affecting combat readiness. This study aimed to identify the clustering patterns and number of HOHCBs in army personnel in Central Peninsular Malaysia. Thus, a cross-sectional study using a multistage sampling technique and a validated 42-item online questionnaire was conducted to assess ten health (medical screening, physical activity, sedentary lifestyle, smoking status, alcohol consumption, substance abuse, aggressive behaviours, sleep, and road safety habits) and five oral health behaviour domains (tooth brushing, fluoridated toothpaste use, flossing, dental visits, and bruxism). Each HOHCB was dichotomised into healthy and health-compromising behaviour and analysed using hierarchical agglomerative cluster analysis (HACA). With the majority being males (92.5%), of other ranks (96.8%), and healthy (83.9%), 2435 army members of a mean age of 30.3 years (SD = 5.9) participated, with a response rate of 100%. HACA identified two clustering patterns: (i) ‘high-risk behaviours’ (30 HOHCBs) and (ii) ‘most common risk behaviours’ (12 HOHCBs) with a mean clustering number of 14.1 (SD = 4.1). In conclusion, army personnel in Central Peninsular Malaysia displayed 2 broad HOHCB clustering patterns, ‘high-risk’ and ‘most common risk’, with an average of 14 HOHCB clusters per person.

## 1. Introduction

The health readiness of military personnel is one of the core sub-factors that directly contribute to ‘combat readiness’, which refers to the military’s preparedness and ability to perform during military operations [1,2]. One of the primary concerns and critical factors that may significantly impact the health readiness of military personnel is health-compromising behaviours [3,4].

Health-compromising behaviours, also referred to as health risk behaviours, are defined as detrimental actions that increase the likelihood of illness or impede recovery [5]. For example, tobacco use, excessive alcohol consumption, physical inactivity, and an unhealthy diet are health-compromising behaviours known to be significant risk factors for cardiovascular diseases, chronic respiratory diseases, cancer, and diabetes. These non-communicable diseases (NCDs), also known as chronic diseases, have become global health problems, as they account for increased health morbidity and mortality worldwide [6]. When coupled with poor oral hygiene practices, these health-compromising behaviours are also known as oral health-compromising behaviours. Evidence has shown that both health- and oral health-compromising behaviours (HOHCBs) negatively affect oral health and overall health, increasing the risk of NCDs [7,8]. More importantly, NCDs such as diabetes mellitus, cardiovascular diseases, chronic respiratory diseases, obesity, and oral diseases (e.g., dental caries, periodontal disease, and oral cancer) directly or indirectly share several behavioural risk factors and intermediary determinants [9,10]. 

These risk factors may not occur in isolation; instead, they commonly co-occur or cluster together [11,12]. For example, a systematic review only focusing on United Kingdom studies reported strong evidence of behavioural clustering of alcohol consumption, smoking, and unhealthy diet in general adult populations [11]. In Spain, three cluster groups, namely, ‘cluster 1: unhealthy lifestyle with moderate risk’, ‘cluster 2: unhealthy lifestyles with high-risk’, and ‘cluster 3: healthy lifestyles with low risk’, comprising seven health-related lifestyles (i.e., smoking, alcohol consumption, drug abuse, physical activity, sedentary habits, dietary habits, and diet quality) were identified in adults [13]. Meanwhile, a study conducted in military personnel assessing the clustering of lifestyle factors in the Hungarian Defence Forces discovered sixteen distinct profiles, including eating habits, smoking status, daily physical activity, sports habits, mental toughness, psychosomatic symptoms, and sleep apnoea symptoms [14]. Other studies related to the clustering of HOHCBs were mainly conducted in the adolescent population [12,15,16,17,18]. For example, a study performed in Saudi Arabian adolescents found two clusters of HOHCBs, namely, non-adherence to preventive behaviours and the undertaking of risk behaviours, clustering among nine health behaviours and three oral health behaviours (i.e., dietary intake, physical activity, sedentary behaviour, smoking status, alcohol consumption, drug use, physical fighting, bullying, use of electronic media communication, frequency of tooth brushing, use of fluoridated toothpaste, and flossing behaviour) [15,16]. Similarly, a study performed in Brazilian adolescents found that the first cluster reflected a combination of the lack of adherence to preventive behaviours and the undertaking of risky conduct, and the second cluster reflected an unhealthy lifestyle [17].

Most importantly, HOHCBs may ultimately impact combat readiness, particularly the health readiness of military personnel. This may result in decreased performance and fitness, affecting military personnel’s physical and mental health, which may later contribute to non-combat injuries (e.g., NCDs) and combat casualties [19,20]. Therefore, determining whether HOHCBs cluster together in military personnel could help to further understand and facilitate prevention, which could benefit combat readiness. In addition, it helps identify the military populations at the greatest risk to plan targeted health promotion and intervention programmes. These initiatives should focus on multiple behaviours, which promises a more significant impact on public health than conventional interventions focusing on a single behaviour, for example, through the common risk factor approach [10]. As such, further evidence, insights, and understanding regarding the occurrence of HOHCBs and how these may cluster and influence the health readiness of army personnel is of paramount importance and very valuable.

To our knowledge, no local study has been conducted on the clustering of HOHCBs in Malaysian adults, and limited studies have been performed in military personnel. The only research study on the clustering of HOHCBs in Malaysia focused on Malaysian adolescents [21]. In addition, previous studies in this area focused on a limited number of behaviours, such as dietary habits, smoking, alcohol consumption, and physical activity. This research gap can limit the understanding of how the diverse health and oral health behaviours are connected and may influence the military population’s health readiness. Therefore, the objective of this study was to identify the clustering patterns and to assess the clustering number of HOHCBs in army personnel in Central Peninsular Malaysia.

## 2. Materials and Methods

### 2.1. Study Design and Sample

A cross-sectional study was conducted in army personnel in an army division of Central Peninsular Malaysia. Using the G*Power 3.0.10 programme, with the power of the study set to 80%, the significance level of 0.050, the dropout rate of 10%, and the design effect of 1.2 [16,21], the sample size required for this study was found to be 1768. Thus, the proposed minimum sample size for the study was 1800. 

A multistage sampling method involving stratified, proportionate, and simple random sampling was employed in different stages. First, the army division, which was divided into four main army troop/brigade groups, was further stratified into four subgroups: Headquarters, Combat Elements, Combat Support Elements, and Services Support Elements. Subsequently, the required sample was divided proportionately according to the ratio of army personnel between each group and subgroup. Each subgroup’s randomly chosen army units were further stratified into officer and other rank groups. After each unit gathered its army personnel, those who fulfilled the inclusion and exclusion criteria within each rank group were invited as the research respondents. The inclusion criteria were all regular (professional) army personnel in service. Those on the resettlement list and outside the unit, training/studying in military/non-military institutions, on active duty, on long vacation, and on sick leave during the data collection period, along with Regular Reserves and Volunteer Force army personnel, were excluded. Based on the ratio, the number of respondents who participate should achieve the minimum number required by the researcher. In this study, all randomly selected army units satisfied the number of respondents needed; thus, the selection of other units was unnecessary.

### 2.2. Study Instrument

The validated online self-administered questionnaire (Google Forms) used in this study was developed and customised based on several existing validated questionnaires used in eleven studies (345 items) measuring health and oral health behaviours [21,22,23,24,25,26,27,28,29,30,31]. Only relevant questions were customised to address the objectives of the present study. The content was validated by the expert committee on the HOHCB questionnaire for Malaysian military personnel. Following a thorough review of the literature and several discussions among members of the expert committee, ten health behaviour domains (‘medical visit’, ‘physical activity’, ‘sedentary lifestyle’, ‘dietary intake’, ‘smoking’, ‘alcohol consumption’, ‘drug and substance abuse’, ‘sleep’, ‘aggressive behaviour’, and ‘road safety behaviour’) and five oral health behaviours (‘toothbrushing’, ‘fluoridated toothpaste’, ‘flossing’, ‘dental visit’, and ‘bruxism’) were identified to be of great importance to military personnel and Malaysian Armed Forces (MAF). Therefore, the HOHCB items were either adopted fully, developed as new items, or modified to fit the military circumstances by rewording, combining, and excluding irrelevant items. 

The questionnaire was pre-tested on 20 army members to assess its face validity, including whether they understood the purpose of the questionnaire, its instructions, the meaning of the items, and the adequacy of the response options, as well as whether they had other related comments. A pilot study involving 248 military members assessing the validity and reliability of the questionnaire revealed a Cronbach’s alpha of acceptable reliability (α = 0.800), with the α-value of all items ranging from 0.300 to 0.980. The factor analysis revealed 16 factors with acceptable Kaiser–Meyer–Olkin (KMO) sampling adequacy values (KMO = 0.740) and factor loadings ranging from 0.303 to 0.968. The questionnaire redistributed to a group of respondents (n = 20) after ten days for test–retest reliability assessment reported a good weighted kappa value for health and oral health behaviours (K = 0.72, *p* < 0.010) and excellent intraclass correlation coefficient (ICC = 0.900, 95% CI (0.880–0.910), *p* < 0.010).

After considering the pre-test and pilot study findings and items essential from military and public health viewpoints, the expert committee agreed on a total of 52 health behaviour items and 9 oral health behaviour items in the final questionnaire. Regarding the sociodemographic background, data included military profile (rank, type of service, and years of service) and general profile (date of birth, gender, ethnicity, marital status, number of children, education level, and medical history), and the respondents’ socioeconomic status was assessed based on their family monthly income [32].

Finally, the self-administered questionnaire comprised ten health-compromising behaviours and five oral health-compromising behaviours. Several items were grouped under the same behaviour; thus, for determining the clustering of HOHCBs, the 42 most relevant items were selected to represent the domains of HOHCBs. Details of each domain are explained in the subsequent sub-sections. 

#### 2.2.1. Health-Compromising Behaviours (10 Domains; 33 Selected Items)

(i) Medical visits (1 item)

The respondents were asked about the frequency of visiting a medical doctor or medical staff for health check-ups or screenings. The response options included ‘12 months or less’, ‘13 months to two years’, and ‘more than two years. Those with more than twelve-month intervals between medical visits were considered to engage in health-compromising behaviour [33].

(ii) Physical activity (1 item)

Six questions elicited the respondents’ frequency of performing three forms of physical activity in the past seven days: (i) vigorous physical activity, (ii) moderate physical activity, and (iii) walking activity. The response was assessed by day (from zero to seven days) and duration (minute). 

The metabolic equivalent task (MET) minutes per week (MET-min week^−1^) were calculated as minutes of [activity/day] × [days per week] × [MET level] [34]. Respondents who had either (i) 75 min of vigorous physical activity, (ii) 150 min of moderate physical activity, or (iii) 600 min of MET of a combination of vigorous, moderate, and/or walking activities in the past seven days were considered to engage in healthy behaviour [28,35].

(iii) Sedentary lifestyle (3 items)

The respondents were prompted to recall the amount of time (hours) that they spent sitting or lying at work (e.g., using a computer), at home (e.g., watching television), during free time (e.g., chatting with friends), and while travelling (e.g., commuting to and from work), and the amount of screen time (hours) spent in both work and daily personal situations. The response options for both questions were ‘one hour or less’, ‘more than one hour to two hours’, ‘more than two hours to four hours’, ‘more than four hours to six hours’, ‘more than six hours to eight hours’, and ‘more than eight hours’. 

A sedentary lifestyle refers to individuals who spend more than eight hours per day sitting and lying [36,37,38], as well as more than two hours of screen time per day using electronic devices. Those with a sedentary lifestyle are considered to engage in health-compromising behaviour [39].

(iv) Dietary intake
  In this domain, those respondents who did not comply with the general recommendations of intake by Malaysia Pyramid Food 2020 and other public health recommendations were considered to display health-compromising behaviours. The items were as follows:
  (a) Plain water (1 item)
    Respondents were asked about the amount of plain water they drank daily, with options ranging from ‘zero’ to ‘eight glasses or more’. Those whose daily plain water intake was less than six glasses were regarded as engaging in health-compromising behaviour [28].
  (b) Major food groups (5 items)
    The respondents were asked about their regularity in consuming (i) fruits, (ii) vegetables, (iii) rice/noodle/bread/cereals/cereal products/tubers, (iv) milk/dairy products, and (v) fish/poultry/meat/eggs/legumes daily, from ‘zero’ to ‘six servings or more’.
    Those consuming less than two servings of fruit, three servings of vegetables, or less than five servings of fruits and/or vegetables per day were regarded as having inadequate dietary intake, thus engaging in health-compromising behaviour [28].
    Respondents who consumed three to five servings of rice/noodle/bread/cereals/cereal products/tubers, two servings of milk and/or dairy products and four servings of poultry/fish/meat/legumes per day were considered to practise healthy behaviour. On the other hand, those who ate less or more were regarded as engaging in health-compromising behaviour [28].
  (c) Sugary foods and beverages, carbonated drinks, and acidic foods (4 items)
    Regarding (i) sugary foods, (ii) sugary beverages, (iii) carbonated drinks, and (iv) acidic foods, respondents were questioned about their daily eating and drinking habits during mealtime with the option to select multiple responses. The response options were ‘breakfast’, ‘morning break’, ‘lunch’, ‘tea time’, ‘dinner’, and ‘supper’. Total sugar intake frequency was calculated by adding the respondents’ frequency of consuming sugary food or drinks over six meals. Those who consumed sugary foods and beverages more than four times a day were deemed to be engaged in health-compromising behaviour, and vice versa [21].
  (d) Fatty food and fast-food intake (3 items)
    Fatty food refers to (i) fried food and (ii) food containing coconut milk (*santan*). Respondents were asked about the frequency of consuming fatty food in the past seven days. The response options were ‘none’, ‘one to two times’, ‘three to four times’, and ‘all seven days’. Those who consumed these foods between three to four times per week or more were deemed to engage in health-compromising behaviour. Respondents were asked about their frequency of (iii) fast-food intake in the past seven days. The response options ranged from zero to seven days.

(v) Smoking (3 items)

Respondents were asked if they had smoked (i) a cigarette and (ii) other forms of tobacco products (i.e., electronic cigarette, shisha/hookah, snuff/chew tobacco, cigar, or other) in the past 30 days and the (iii) frequency of being exposed to tobacco product smoke in the previous week. The respondent who smoked any tobacco products on one or more days in the past 30 days was considered a ‘current smoker’ and thus to engage in health-compromising behaviour, while those who had been exposed to any tobacco product smoke for at least one day were considered to engage in health-compromising behaviour.

(vi) Alcohol consumption (1 item)

The question regarded the frequency of drinking at least one alcoholic beverage in the past 30 days. The respondent was considered a ‘current drinker’ if they had consumed alcoholic drinks on one or more days in the past 30 days, and they were categorised as displaying health-compromising behaviour [28].

(vii) Drug and substance abuse (1 item)

Any illegal use of substances such as opioids (e.g., marijuana, heroin, and morphine), amphetamine or methamphetamine (e.g., ecstasy, syabu, ice, and yaba pills), kratom (*ketum*), and inhalants (e.g., glue and petrol) was defined as drug and substance abuse [28]. It also included the abuse and misuse of prescription medicines, such as painkillers, cough syrup, or sleeping pills [27]. 

The respondents were asked whether they had been using any forms of drugs or engaged in substance abuse. The response options ranged between ‘Never’, ‘Yes, for the past 30 days’, and ‘Yes, in the entire life but not the past 30 days’. Respondents who had abused drugs or substances at least once in the past 30 days were categorised as current drug users practising health-compromising behaviour [28].

(viii) Sleep behaviour (3 items)

The respondents were asked about their sleeping hours (i) during the working week and (ii) on weekends or holidays, as well as the amount of time that they required to feel refreshed and to function normally (ranging from ‘four hours or less’ to ‘more than eight hours’), and (iii) the perceived quality of sleep in the past seven days (‘excellent’, ‘good’, ‘moderate’, and ‘poor’). Respondents whose daily sleeping hours were equal to or greater than their perceived hours to feel refreshed and function well engaged in healthy behaviour, while others were deemed to engage in health-compromising behaviour [27].

(ix) Aggressive behaviour (1 item)

Aggressive behaviours included yelling or shouting, kicking or smashing objects, threatening with physical violence, and physically fighting or hitting an individual in the past 30 days. The choices of response ranged from ‘none’ to ‘five times or more’. Respondents who had been involved in any form of aggressive behaviour for one or more days in the past 30 days were considered to engage in health-compromising behaviour [27].

(x) Road safety behaviour (6 items)

Respondents were asked about their frequency of wearing (i) seat belt and (ii) helmet, as well as (iii) texting and (iv) calling others while driving or riding a vehicle in the past seven days. The questions on the seat belt and helmet concerned the driver/rider and passenger. The response options were ‘always’, ‘most of the time’, ‘seldom’, ‘never’, and ‘I did not drive or ride a vehicle in the past seven days’. Respondents who claimed that they ‘always’ wore a seatbelt and helmet and ‘never’ texted or called while driving or riding a vehicle in the past seven days were determined as practising healthy behaviour, and vice versa [27].

#### 2.2.2. Oral Health-Compromising Behaviours (5 Domains; 9 Items)

(i) Tooth Brushing (2 items)

Respondents were asked about the frequency of brushing their teeth and the time spent tooth brushing daily. Answer options ranged from ‘rarely’, ‘less than three times a week’, ‘three to four times a week’, ‘once a day’, and ‘twice a day or more’, and ‘after waking up from sleep’, ‘before breakfast’, ‘after main meals’, ‘after eating sugary food or drink’, and ‘before sleep’. Those who brushed their teeth less than twice a day and did not brush before sleep were considered as displaying oral health-compromising behaviours.

(ii) Toothpaste (2 items)

Respondents had to indicate whether their toothpaste was ‘fluoridated’, ‘not fluoridated’, ‘not sure’, or ‘I did not even know what fluoride was’ and subsequently state the brand of their toothpaste. Those who used non-fluoridated toothpaste were considered as practising oral health-compromising behaviours.

(iii) Flossing (1 item)

Respondents were asked about the frequency of flossing in the past 30 days. The response options were ‘once daily’, ‘once every two days’, ‘once to twice a week’, ‘once to twice a month’, ‘rarely or never’, and ‘I do not know floss’. Those who flossed less than once every two days were considered as practising oral health-compromising behaviours.

(iv) Dental visits (3 items)

Respondents had to indicate the time interval since their (i) last dental visit, whether it was ‘6 months or less’, ‘more than six months to one year’, ‘more than one year to 2 years’, or ‘more than two years’. In addition, respondents had to state the main reason for their most recent dental visit, both on (ii) their initiative or (iii) as a part of service requirements. The response options were ‘dental check-up’, ‘preventive treatment’, ‘treatment of related oral health problems’, and ‘I did not visit the dentist in the past 12 months’.

Those who had had their dental check-up more than 12 months before and whose last dental visit was for oral health problem treatment, or those who had not visited the dentist in the past 12 months were considered as practising oral health-compromising behaviours.

(v) Bruxism (1 item)

This question elicited the respondents’ awareness of clenching and grinding their teeth while asleep as told by their spouse or family members. The response options were either ‘Yes ‘or ‘No’, and those with bruxism had oral health-compromising behaviours.

### 2.3. Conduct of the Study

The authors obtained ethical permission from relevant authorities, including Universiti Malaya and MAF, before conducting the study. Once approval was obtained, an online briefing was held for the person in charge (PIC) of the army units via Google Meet. In addition, face-to-face briefings were conducted with units that could not attend the online session.

The questionnaire was administered to army personnel of Central Peninsular Malaysia units. The briefing session (physical, online, or video briefing) and questionnaire distribution were conducted according to the availability of each unit. Those who agreed consented to participate in the study. The respondents then scanned a quick response (QR) code or visited a website link to a Google form via their smartphone or personal computer. While answering the survey questions, a researcher was present (or in a WhatsApp group/personal message/call) to help the respondents if they faced any questions or problems. After the respondents completed the questionnaire, the researchers checked each questionnaire to ensure all sections were completed and submitted. Any incomplete questionnaires were rectified by informing the respondents (or through PICs) and asking them to complete their attempts.

### 2.4. Data Management

In order to protect the confidentiality of the online data, the Google documents were password-protected and deleted from the ’cloud‘ after they were downloaded. Only the researchers had access to the password-protected documents stored on the external hard drive. Each respondent was given an individual identifier, and their names and Army Identification Numbers were not utilised in any data analysis or presentation.

There were 6.4% of respondents who had at least one missing value, where the proportion of items with a missing value ranged from 1.4% to 5.4%. No respondents were excluded from the study because the missing value was far less than the 20% cut-off point. Subsequently, in the data analysis, we used mean values (unit imputation) to replace the missing values. Therefore, the completion rate was 100% (N = 2435).

All data were entered into Statistical Package for Social Sciences (SPSS) software, version 25. They were initially checked, cleaned, and explored using descriptive statistics and graphs for each variable. Next, means and standard deviations (SDs) were used to describe the continuous variables, while frequencies and percentages were used for categorical data. The data on each HOHCB were then dichotomised into binary codes (0 = healthy behaviour; 1 = health-compromising behaviour) for the 42 HOHCB items (Table S1).

### 2.5. Statistical Analysis

Hierarchical agglomerative cluster analysis (HACA) with the between-groups linkage clustering method and squared Euclidean distance measure without pre-determined cluster membership was used in the analysis. HACA was chosen because it allows researchers (i) to compare the clustering result with an increasing number of clusters, (ii) because there is no need to make an a priori decision about the final number of clusters, and (iii) because it is more stable than the non-hierarchical method [40]. 

Two-step HACA with the between-group linkage clustering method and squared Euclidean distance measure without pre-determined cluster membership were performed in this study. The first step was to generate a squared Euclidean proximity matrix of all 42 HOHCB items, while the second step involved the use of a dendrogram to visualise the distance and cluster combination. The number of clusters was counted and reported as frequency and percentage, including the mean number of clustering. 

In addition, repeated HACA on different sub-samples and K-means cluster analysis were used to validate the findings compared to the two-step HACA findings.

## 3. Results

### 3.1. Sociodemographic Characteristics

As shown in Table 1, the mean age of all the 2435 army members (response rate = 100%) was 30.3 years (SD = 5.9), with the majority being male (92.5%). In addition, most respondents were of Malay ethnicity (77.0%), married (67.5%), had received education up to the secondary school level (87.4%), and had no medical conditions (83.9%).

Military-wise, almost all respondents were army personnel of other ranks (96.8%), with two-thirds of them being junior non-commissioned officers (NCOs) (66.2%). Nearly 60% of them had served for 12 years or less. According to the respondent’s Corps and Regiments, less than half of them were from the Combat Element (45.3%), and approximately one-fourth were from the Combat Support Element (28.9%), while the remaining quarter was from the Services Support Element (25.7%). Socioeconomically, the vast majority of respondents were in the bottom 40% (B40; MYR 4849 and below) family income group (92.6%).

### 3.2. Clustering Patterns of HOHCBs

#### 3.2.1. Hierarchical Agglomerative Cluster Analysis

Euclidean distances were calculated between all pairs of cases, where a low coefficient indicated high similarity between the two variables, thus classified as one group. Conversely, a high coefficient indicated low similarity between the variables, and they became more heterogeneous than the previous combinations (Table S2).

**Figure 1:**
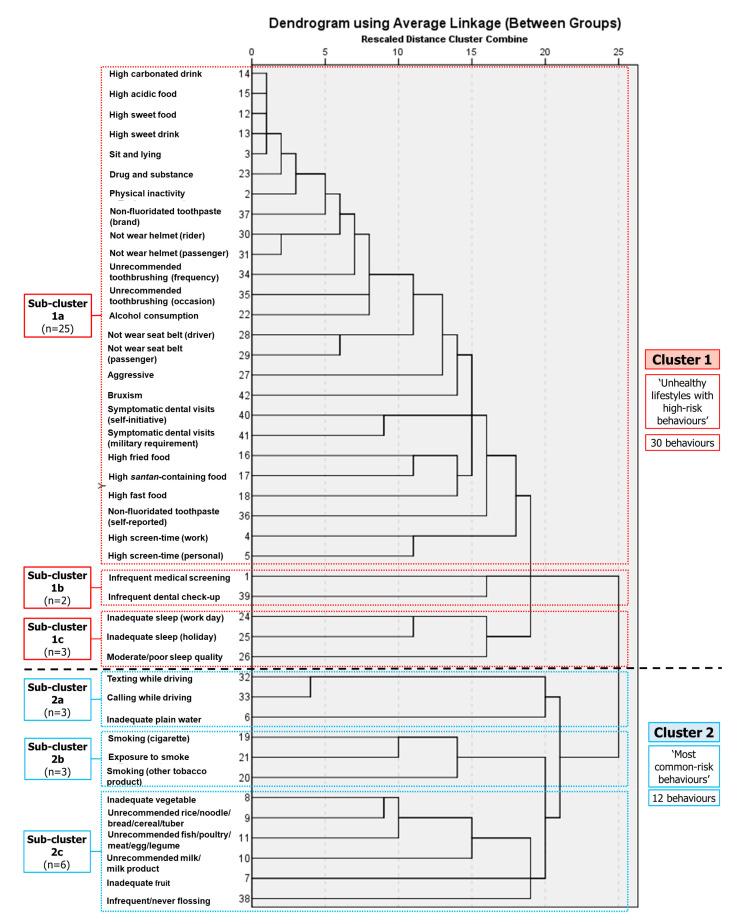
Dendrogram of hierarchical agglomerative cluster analysis of the 42 health- and oral health-compromising behaviours.

As shown in Figure 1, the cluster began with the smallest squared Euclidean distance of high consumption of carbonated drinks and acidic foods (9.000). Subsequently, the cluster was combined with high sweet-food consumption, high sweet-drink consumption, and sitting and lying behaviours to form a small group, which merged with other behaviours and groups, forming Sub-cluster 1a (1001.348). This sub-cluster consisted of 25 behaviours (high consumption of carbonated, acidic, and sweet foods and drinks; extended sitting and lying; drug use; physical inactivity; non-fluoridated toothpaste use; not following tooth-brushing recommendations; alcohol consumption; not wearing a helmet and seat belt; aggressive behaviour; bruxism; symptomatic dental visits; high fatty-food consumption; and extended screen time). Meanwhile, another smaller group comprising two behaviours, namely, medical screenings and dental check-ups, formed Sub-cluster 1b (845.000). Next, inadequate sleep during work and holidays formed a cluster (584.000) combined with substandard sleep quality to form Sub-cluster 1c (886.000). Subsequently, Sub-cluster 1a was merged with Sub-cluster 1b (1043.580) and Sub-cluster 1c (1065.741). Finally, these three sub-clusters (Sub-clusters 1a + 1b + 1c) were combined to form a broad cluster, Cluster 1.

At the bottom of the dendrogram are Sub-clusters 2a, 2b, and 2c. Both Sub-cluster 2a (low plain-water consumption, and texting and calling while driving) (1072.000) and Sub-cluster 2b (exposure to smoke, and smoking cigarettes and other tobacco products) (744.500) had three behaviours, respectively. Sub-cluster 2c had six behaviours (unrecommended vegetable, fruit, cereal/cereal-product, milk/dairy-product, and poultry/fish/meat/legume consumption; and infrequent flossing) (1001.500). Sub-cluster 2a was subsequently merged with combined Sub-cluster 2b and Sub-cluster 2c (1111.944) to form a broad cluster, Cluster 2 (Sub-clusters 2a + 2b + 2c).

Cluster 1 was named ‘unhealthy lifestyles with high-risk behaviours’ and comprised 30 HOHCBs. Cluster 2 was named ‘most common risk behaviours’ and contained 12 HOHCBs. At the end of the analysis, Cluster 1 and Cluster 2 were merged into one large cluster (1399.217). The validity and stability of the cluster analysis were confirmed by performing repeated HACA on different sub-samples randomly drawn from the study sample (SPSS random sample of cases; approximately 25% of respondents for each sample) [12,16].

#### 3.2.2. K-Means Cluster Analysis

K-means cluster analysis requires an a priori number of clusters to be extracted from the data before performing the analysis. Therefore, using information obtained with the HACA, K-means cluster analysis with two cluster memberships was used. As a result, Cluster 1 comprised 50.3% (n = 1224) of respondents. Regarding the average score (mean) for each HOHCB, the cluster consisted of 17 HOHCBs. These included infrequent medical screenings; extended screen time (work); extended screen time (personal); low fruit consumption; high consumption of fried food; inadequate sleep during working days; flossing infrequently/never flossing; infrequent dental check-ups; symptomatic dental visits upon self-initiative; low vegetable consumption; unrecommended rice/noodle/cereal/tuber, milk/dairy-product, and poultry/fish/meat/egg/legume consumption; cigarette smoking; exposure to cigarettes/tobacco products smoke; and texting and calling while driving. In contrast, Cluster 2 comprised 49.7% of respondents, with nine HOHCBs. Eight were the same as in Cluster 1, with one distinct behaviour, low plain-water consumption. In addition, there were 24 HOHCBs with a mean score equal to zero for both cluster memberships (Table S3).

### 3.3. Clustering Number of HOHCB

The clustering HOHCB number for each respondent was calculated, and the total score was interpreted as the clustering HOHCB number for all respondents. The clustering number for the 42 behaviours ranged from 0 (no HOHCBs) to 29 HOHCBs, with the average number of clustering behaviours in army personnel being 14.1 (SD = 4.1). The most common clustering number among the respondents was 15 (9.2%), followed by 13 (9.0%), 16 (8.7%), and 14 (8.7%) HOHCBs (Table 2).

## 4. Discussion

As this is the first study investigating HOHCB clustering in this population in Malaysia, this study investigated multiple important and relevant HOHCBs in the adult and MAF populations, consisting of 15 HOHCB domains (health behaviours: 10 domains, with 33 items; oral health behaviours: 5 domains, with 9 items), for a total of 42 items. In comparison with other studies with a lesser number of items, ranging from 2 [41] to 17 items [17], conducted in adolescents and adults in the United Kingdom, the United States, Brazil, Saudi Arabia, Malaysia, and Korea, the present study considered a large number of HOHCB items, including most HOHCB domains from previous studies. The reason was to allow us to conduct a complete and all-inclusive assessment of all relevant HOHCBs in the MAF population to reflect the actual situation and use the information to advocate for comprehensive health and oral health programmes.

HACA and K-means cluster analysis revealed two broad clusters in respondents with a relatively similar pattern. Nevertheless, one cluster membership contained more HOHCBs than the other, reflecting a high-risk behaviour clustering group. The first cluster, ‘high-risk behaviours’, consisted of 30 HOHCBs. The cumulative and synergistic negative effects of health-compromising behaviours increase the risk of adverse effects on health [5,42]. Hence, the respondents in this group may have a higher chance of developing diseases, with those diseases having a higher chance of having greater severity. Physical inactivity and sedentary lifestyles, as well as fast food, fatty-food, and high sugar intake, contribute to increased risk of obesity and overweight, cardiovascular diseases, musculoskeletal disorders, and cancer [43,44]. Furthermore, both drug abuse and alcohol harm health and functional ability. They may also have medical (e.g., addiction, immunodepression, infectious diseases, and mortality), physical (e.g., physical abuse and injuries, agitation, and restlessness), social (e.g., crime, interpersonal and relationship problems, and loss of employment), and psychological (e.g., mental illness, violent and aggressive behaviour, and suicidal behaviour) implications. Meanwhile, sleep deprivation increases the risk of depression, suicide, post-traumatic stress disorder, accidents and injuries, cardiometabolic disorders, and even mortality [45]. The risk of major road traffic injuries, including thoracic, head, and neck injuries, and even mortality is increased when seatbelts and helmets are not used [46,47]. Moreover, unrecommended tooth-brushing frequency, non-fluoridated toothpaste use, and symptomatic dental visits are associated with oral health diseases such as dental caries, periodontal disease, and edentulousness, all of which negatively impact the quality of life [48,49].

The second cluster comprised a total of 12 HOHCBs, involving respondents who displayed (i) ‘unhealthy nutrition intake’ (unrecommended cereal and cereal-product consumption; unrecommended fish, poultry, meat, egg, and legume consumption; unrecommended milk and dairy-product consumption; low fruit intake; and low plain water intake), (ii) ‘tobacco product use’ (smoking cigarettes, use of other tobacco products, and exposure to tobacco product smoke), (iii) ‘risky driving’ (texting and calling while driving), and (iv) flossing infrequently or never flossing. Most of these HOHCBs were among the eleven most prevalent health-compromising behaviours in the army personnel in this study. At least two-fifths of respondents engaged in these HOHCBs (44.2% (other tobacco product use)–82.0% (unrecommended cereal and cereal-product consumption)). Thus, the second cluster may represent the most common HOHCBs in army personnel. The possibility of adverse health impacts also exists in the second cluster group. For instance, malnutrition, micronutrient deficiencies, anaemia, gastrointestinal diseases, and osteoporosis have all been linked to unhealthy dietary consumption [50,51]. Tobacco products (e.g., cigarettes and electronic cigarettes) and second-hand smoke are associated with several adverse health effects, such as respiratory diseases, cardiovascular diseases, and cancer, all of which contribute to premature mortality [52,53]. Texting and calling without a hands-free device while driving endanger drivers, passengers, and other road users, as these behaviours increase the likelihood of accidents due to the driver’s inability to focus on the road, respond to major traffic events, and maintain vehicle control within the lane [54,55]. Flossing infrequently or never flossing is associated with an increased risk of interproximal caries and periodontal disease [56]. 

However, no direct comparison can be made between the present study and previous studies of clustering HOHCBs, given that the present study comprised a different number of clustering patterns with varying HOHCB composition. In previous studies, the number of clustering patterns ranged from two [12,16,17,21,57] to six clusters [58]. A study in the Hungarian Defence Forces personnel reported 16 cluster profiles, but the clusters were also classified according to military characteristics [14].

Nevertheless, the present study’s findings are comparable in terms of ‘risk categorisation’. In this study, the clustering patterns in army personnel were categorised into ‘high-risk behaviours’ and ‘most common risk behaviours’, a type of categorisation similar to that in studies in Europe, China, Australia, the United Kingdom, and the United States [13,38,57,59,60,61,62]. For instance, Hobbs et al. [38] identified three clusters, (i) ‘lower risk’, (ii) ‘moderate risk’, and (iii) ‘elevated risk’ in Australian adults. A United Kingdom study conducted in adults by Mawditt et al. [60] also performed similar categorisations: (i) ‘risky’, (ii) ‘moderate’, and (iii) ‘mainstream’ clustering pattern groups. These studies classified the clustering patterns as ‘high-risk’ and ‘low/moderate/mainstream’, where mainstream behaviour is similar to the most common risk behaviour. Additionally, a study performed in Hong Kong adults by Chan and Leung [57] identified two clusters: (i) ‘healthy’ and (ii) ‘less healthy’. This risk categorisation was based on the type and number of risk behaviours in the cluster. It determined the level of the clustering group in relation to the likelihood of developing diseases and the severity of diseases. A greater number of HOHCBs, for example, increase the risk of disease. Furthermore, categorisation may aid in determining which clusters to focus on, such as the targeted population group and high-risk behaviour approach in health promotion activities [10,12].

At the same time, some compromising behaviours clustered together within the same cluster group. Similar to Cluster 1 (‘high-risk behaviours’) in the present study, Ali [21] reported that alcohol consumption, aggressive behaviours (bullying and physical fighting) and drug users clustered in the same group in Malaysian adolescents. In addition, Jordao, Malta, and Freire [17], and Skalamera and Hummer [62] reported that alcohol consumption clustered with drug use in young adults in the United States and Brazil. In terms of sedentary lifestyles and unrecommended dietary intake, Hobbs et al. [38] revealed that physical inactivity, extended sitting time, and fast-food consumption clustered within the ‘elevated risk’ cluster. A similar pattern was reported by Skalamera and Hummer [62], where physical inactivity and fast-food consumption clustered together, but with the addition of no doctor or dentist visits. Regarding Cluster 2 (most common risk behaviours), the results align with the findings by Ali [21], in which inadequate consumption of vegetables and fruits, and low consumption of milk and dairy products clustered in a group.

Identifying the two HOHCB cluster patterns in army personnel, as conducted in the present study, is essential for oral health promotion activities. The findings assist in better understanding risk factor clustering and could facilitate prevention, which could benefit the combat readiness of military populations. Thus, health promotion and intervention programmes could be prioritized and target high-risk personnel. These initiatives should focus on multiple behaviours, which promises a more significant impact on public health than conventional interventions focusing on a single behaviour, for example, through the common risk factor approach. For instance, in the population-based approach, health promotion activities should focus on behaviours in the ‘Cluster 2: most common risk behaviours’ clustering pattern, since they involve several of the most common HOHCBs in army personnel. The goal is to empower army personnel regarding proper oral hygiene self-care (particularly, flossing), healthy nutrient consumption, smoking control programmes, and road safety from a health standpoint. Therefore, tackling this behaviour cluster may help reduce the prevalence of the most common risk behaviours in army personnel. Nevertheless, in a situation where resources are available, targeting the first cluster (high-risk behaviours) has its advantages. As the cluster consists of 30 health-compromising behaviours, addressing this behaviour cluster might eventually tackle many HOHCBs in the army.

Equally important is the number of clustering HOHCBs that can occur in one army member. This study revealed that on average, army personnel could have 14.1 (SD = 4.1) clustering behaviours, with the most common clustering number in the respondents being 15 (9.2%), followed by 13 (9.0%), 16 (8.7%), and 14 (8.7%) HOHCBs. However, the results cannot be compared with those of other studies, because studies on clustering numbers are minimal, with different numbers of HOHCBs being investigated. Nonetheless, this could be one of the novelties of the present study in terms of clustering behaviour research, as the findings prove that an individual can simultaneously engage in multiple HOHCBs. For example, on average, one army member in this study engaged in 14 HOHCBs, which is alarming. Knowing the clustering number could also be the focus of prevention and promotion activities, prioritising those with a higher number of clustering risk behaviours. Additionally, identifying the clustering number could complement the use of information on clustering patterns. For example, during the planning and execution of an intervention, we could concentrate on one clustering pattern and target those with high clustering numbers within the cluster. Hence, it is highly recommended that future studies investigate the clustering patterns and clustering number of HOHCBs at the individual level.

This study has a few limitations. First, the results heavily depend on the respondents’ truthfulness in the use of the self-administered questionnaire. Some HOHCB items might have been under-reported or over-reported, e.g., substance abuse and alcohol consumption. Second, the present study only involved army personnel in Central Peninsular Malaysia. Similar data on the navy and air force personnel who are also part of the three main MAF branches were not included. They may have different HOHCB clustering patterns. This also includes other army formations such as other infantry divisions and army troops. However, the main strength of the study is that limited studies have looked into clustering patterns and clustering numbers of HOHCBs, especially in military personnel, and this study is one of the few that have done so. Although the findings are here mostly compared with studies in the general population, the results are still beneficial to providing evidence and assisting in reorienting the health promotion approaches in the army population. Thus, more studies on the clustering of HOHCBs in military personnel should be conducted in the future, since the HOHCBs are linked to the health readiness and, by extension, the combat readiness of the MAF.

## 5. Conclusions

This study identified two broad HOHCB clustering patterns in army personnel in Central Peninsular Malaysia, ‘high-risk’ behaviours and ‘most common risk behaviours’, with an average of 14 HOHCB clusters for each army member. As HOHCBs significantly impact the health readiness and, eventually, the combat readiness of the army population, taking cognisance of these findings could assist policy makers or health managers in formulating strategies for effective health promotion and disease prevention programmes targeting the army personnel engaged in these two HOHCB clusters. The common risk factor approach becomes very relevant and could be applied. With limited resources, future health prevention and promotion policies and activities should focus on army personnel engaged in Cluster 2 ‘most common risk behaviours’, as the findings show that this cluster comprises the most prevalent health-compromising behaviours in army personnel, followed by those engaged in Cluster 1 behaviours. Furthermore, policymakers or health managers could further prioritise those with a high number of clustered risk behaviours within this targeted group. These strategies can hopefully tackle the factors that contribute the most to the army population’s health readiness, thus further maintaining the army’s combat readiness.

## Figures and Tables

**Table 1:** Characteristics of study respondents (N = 2435).

Sociodemographic Characteristic	Mean (SD	Frequency (n)	Percentage (%)
Age	30.3 (5.9)		
Gender			
Male		2253	92.5
Female		182	7.5
Ethnicity			
Malay		1876	77.0
Bumiputera Sabah		308	12.7
Bumiputera Sarawak		168	6.9
Indian		49	2.0
Chinese		12	0.5
Others		22	0.9
Marital status			
Single		745	30.6
Married		1643	67.5
Divorced/widow/widower		47	1.9
Level of education			
Secondary school		2128	87.4
Certificate		109	4.5
Malaysian higher school certificate/ matriculation		50	2.0
Diploma		68	2.8
Degree and above		80	3.3
Medical condition ^1^			
No medical condition		2042	83.9
One or more medical conditions		393	16.1
Rank			
Other ranks		2356	96.8
Officer		79	3.2
Corps or Regiment			
Combat Element		1104	45.3
Combat Support Element		704	28.9
Services Support Element		627	25.8
Service duration			
12 years or less		1395	57.3
From 12 to 18 years		863	35.4
More than 18 years		177	7.3
Family income ^2^			
Bottom 40% (B40; MYR 4849 or less)		2256	92.7
Middle 40% (M40; MYR 4850–MYR 10,959)		169	6.9
Top 20% (T20; MYR 10,960 or more)		10	0.4

^1^ Medical conditions included self-reported hypertension, diabetes mellitus, hypercholesterolemia, respiratory conditions, cardiovascular conditions, cancer, and obesity. ^2^ Household income levels in accordance with information from the Malaysian Department of Statistics 2019 report [32].

**Table 2:** Mean and frequency distributions of the clustering count of 42 HOHCBs in respondents.

Clustering Number	Mean (SD) Clustering Number	Frequency (n)	Percentage (%)
	14.1 (4.1)		
0 (all healthy)		0	0.0
1		1	0.0
3		5	0.2
4		4	0.2
5		17	0.7
6		32	1.3
7		54	2.2
8		84	3.4
9		114	4.7
10		173	7.1
11		204	8.4
12		191	7.8
13		219	9.0
14		211	8.7
15 *		223	9.2
16		213	8.7
17		197	8.1
18		136	5.6
19		113	4.6
20		82	3.4
21		52	2.1
22		50	2.1
23		34	1.4
24		9	0.4
25		9	0.4
26		3	0.1
27		3	0.1
28		1	0.0
29		1	0.0
Total		2435	100.0

* Most common clustering number among the respondents.

## Data Availability

The data presented in this study are only available upon request from the corresponding author. They are not publicly available due to privacy and ethical issues.

## Supplementary Materials

The following supporting information can be downloaded at: https://www.mdpi.com/article/10.3390/healthcare11050640/s1, Table S1: Categorisation of healthy and health-compromising behaviours, Table S2: Agglomeration schedule of the average linkage between groups, Table S3: K-means cluster analysis: number of respondents and the final cluster centre of health- and oral health-compromising behaviours.
