# Supplementary material for: Clustering of Health and Oral Health-Compromising Behaviours in Army Personnel in Central Peninsular Malaysia
Source: Healthcare (Basel). 2023 Feb 21;11(5):640. doi: 10.3390/healthcare11050640 (PMC10000684; doi:10.3390/healthcare11050640)
Supplement: Supplementary file 1 [file healthcare-11-00640-s001.zip › healthcare-2171326-supplementary.pdf]

Table S1. Categorisation of healthy and health-compromising behaviours

Item			Category/Response key	
			Healthy behaviour	Health-compromising behaviour
1. Medical visit			12 months or less	More than 12 months
2. Physical activity	Vigorous activity		75 minutes or more per week	Less than 75 minutes per week
	Moderate activity		150 minutes or more per week	Less than 150 minutes per week
	Vigorous + moderate + walking activity		600 METs or more per week	Less than 600 METs per week
3. Sedentary lifestyle	Sitting and lying		8 hours or less per day	More than 8 hours per day
	Screen time		2 hours or less per day	More than 2 hours per day
4. Dietary intake	Plain water		6 glasses or more per day	Less than 6 glasses per day
	Fruits and/or vegetables	Fruits only	2 servings or more per day	Less than 2 servings per day
		Vegetables only	3 servings or more per day	Less than 3 servings per day
		Fruits and vegetables	5 servings or more per day	Less than 5 servings per day
	Rice, noodle, bread/cereals, cereal products/tubers		3 to 5 servings per day	Less than 3 servings per day
				More than 5 servings per day
	Milk/dairy products		2 servings per day	Less than 2 servings per day
				More than 2 servings per day
	Poultry/fish/meat/legumes		3 to 4 servings per day	Less than 3 servings per day
				More than 4 servings per day
	Sugary foods/beverages		4 times or less per day	More than 4 times per day
	Carbonated drinks		3 times or less per day	More than 3 times per day
	Acidic foods			
	Fatty foods	Fried food	Less than 3-4 times/ week	3–4 times/week or more
		Santan-containing food		
	Fast food		1 time or less in the past 7 days	More than 1 time in the past 7 days
5. Smoking	Cigarette/other tobacco product		None	Current smoker. Smoked cigarette or other tobacco products for the past 30 days.
	Exposed to cigarette smoke		Less than 1 day in the past 7 days	1 or more days in the past 7 days
6. Alcohol consumption			None	Drunk at least one alcoholic drink, one or more days for the past 30 days.
7. Drug and substances abuse			None	Any drugs or substance abuse in the past 30 days
8. Aggressive			None	Any aggressive involvement in one or more days for the past 30 days.

Table S1 (continued). Categorisation of healthy and health-compromising behaviours

Item		Categorised/ Response key	
		Healthy behaviour	Health-compromising behaviour
9. Sleep	Hour	Daily sleep hour equivalent to or more than perceived hour to feel fresh and function well.	Daily sleep hour less than perceived hour to feel fresh and function well.
	Quality	Perceived good and excellent sleep quality.	Perceived poor and moderate sleep quality
10. Road safety behaviour	Seat belt, helmet, texting, or call	Always wearing seatbelt and helmet, and never text or call while driving or riding a vehicle in the past 7 days.	Not wearing seatbelt and helmet, texting or calling while driving or riding a vehicle in the past 7 days.
11. Toothbrushing	Frequency	Twice a day or more	Less than twice a day
	Time	Brush two or more times and had 'before sleep'.	Brushing less than two time or no 'before sleep'
12. Toothpaste	Fluoride	Use fluoridate toothpaste	Use non-fluoridated toothpaste
	Brand name		
13. Flossing		Once every two days or more	Less than once every two days
14. Dental visit	Dental check-up	One year or less	More than one year
	Last dental visit	Dental check-up and preventive treatment in the past 12 months.	Treatment for an oral health problem and do not visit the dentist in the past 12 months.
15. Bruxism		No bruxism	Bruxism

Table S2. Agglomeration schedule of the average linkage between groups

Agglomeration Schedule						
	Cluster Combined			Stage Cluster First Appears		
Stage	Cluster 1	Cluster 2	Coefficients	Cluster 1	Cluster 2	Next Stage
1	14	15	9.000	0	0	2
2	12	14	25.500	0	1	3
3	12	13	38.333	2	0	4
4	3	12	57.250	0	3	6
5	30	31	95.000	0	0	10
6	3	23	119.400	4	0	7
7	2	3	168.167	0	6	9
8	32	33	224.000	0	0	38
9	2	37	245.714	7	0	10
10	2	30	304.500	9	5	12
11	28	29	309.000	0	0	21
12	2	34	353.500	10	0	13
13	2	35	403.364	12	0	14
14	2	22	436.417	13	0	21
15	8	9	488.000	0	0	18
16	40	41	502.000	0	0	28
17	19	21	525.000	0	0	24
18	8	11	552.000	15	0	27
19	4	5	568.000	0	0	33
20	24	25	584.000	0	0	32
21	2	28	598.423	14	11	23
22	16	17	605.000	0	0	26
23	2	27	680.467	21	0	25
24	19	20	744.500	17	0	39
25	2	42	765.563	23	0	28
26	16	18	776.500	22	0	29
27	8	10	796.667	18	0	34
28	2	40	811.412	25	16	29
29	2	16	829.947	28	26	31
30	1	39	845.000	0	0	35
31	2	36	884.045	29	0	33
32	24	26	886.000	20	0	37
33	2	4	1001.348	31	19	35
34	7	8	1015.000	0	27	36
35	1	2	1043.580	30	33	37
36	7	38	1056.600	34	0	39
37	1	24	1065.741	35	32	41
38	6	32	1072.000	0	8	40
39	7	19	1111.944	36	24	40
40	6	7	1139.741	38	39	41
41	1	6	1399.217	37	40	0

Table S3. K-means cluster analysis: Number of respondents and the final cluster centre of the health and oral health-compromising behaviours (N=2435)

**K-means cluster analysis**	Cluster				
	1		2		
	n	%	n	%	
Number of respondents (n)	1224	50.3	1211	49.7	
**Final Cluster Centers**					
**Health and oral health-compromising behaviours**	Mean				*p-value*[1]
Infrequent medical screening	1		0		<0.001*
High screen-time (work)	1		0		<0.001*
High screen-time (personal)	1		0		<0.001*
Low fruit	1		0		<0.001*
High fried food	1		0		<0.001*
Inadequate sleep (work day)	1		0		<0.001*
Infrequent/never floss	1		0		<0.001*
Infrequent dental check-up	1		0		<0.001*
Symptomatic dental visit (self-initiative)	1		0		<0.001*
Physical inactivity	0		0		0.242
High sitting and lying down	0		0		<0.001*
High sweet food	0		0		0.578
High sweet drink	0		0		0.284
High carbonated drink	0		0		0.102
High acidic food	0		0		0.21
High *santan*-containing food	0		0		<0.001*
High fast food	0		0		0.001*
Smoking (other tobacco products)	0		0		0.343
Alcohol	0		0		<0.001*
Drug	0		0		0.001*
Inadequate sleep (holiday)	0		0		<0.001*
Sleep substandard quality	0		0		<0.001*
Aggressive behaviours	0		0		<0.001*
Not wearing seat belt (driver)	0		0		<0.001*
Not wearing seat belt (passenger)	0		0		<0.001*
Not wear helmet (rider)	0		0		0.988
Not wear helmet (passenger)	0		0		0.878
Infrequent toothbrushing (frequency)	0		0		0.045*
Unrecommended toothbrushing (occasion)	0		0		<0.001*
Non-fluoridated toothpaste (self-report)	0		0		0.442
Non-fluoridated toothpaste (recheck brand)	0		0		0.376
Symptomatic dental visit (military requirement)	0		0		<0.001*
Bruxism	0		0		<0.001*
Low plain water	0		1		0.002*
Low vegetable	1		1		<0.001*
Unrecommended rice/noodle/bread/cereal/tuber	1		1		0.049*
Unrecommended milk/dairy products	1		1		<0.001*
Unrecommended poultry/fish/meat/egg/legume	1		1		0.024*
Smoking (cigarette)	1		1		0.104
Smoke (exposed)	1		1		<0.001*
Texting while driving	1		1		<0.001*
Calling while driving	1		1		0.048*

[1]*p-value:* (ANOVA): The F tests should be used only for descriptive purposes because the clusters have been chosen to maximise the differences among cases in different clusters. The observed significance levels are not corrected for this and thus cannot be interpreted as tests of the hypothesis that the cluster means are equal.
*Significant at $p < 0.050$.
